# Genome sequence of the marine bacterium *Corynebacterium maris* type strain Coryn-1^T^ (= DSM 45190^T^)

**DOI:** 10.4056/sigs.4057796

**Published:** 2013-07-30

**Authors:** Lena Schaffert, Andreas Albersmeier, Hanna Bednarz, Karsten Niehaus, Jörn Kalinowski, Christian Rückert

**Affiliations:** 1 Technology Platform Genomics, CeBiTec, Bielefeld University, Bielefeld, Germany; 2 Proteome and Metabolome Research, Bielefeld University, Bielefeld, Germany

**Keywords:** aerobic, non-motile, Gram-positive, non-spore forming, non-hemolytic, heterotrophic, mesophilic, halotolerant

## Abstract

*Corynebacterium maris* Coryn-1^T^ Ben-Dov *et al.* 2009 is a member of the genus *Corynebacterium* which contains Gram-positive, non-spore forming bacteria with a high G+C content. *C. maris* was isolated from the mucus of the Scleractinian coral *Fungia granulosa* and belongs to the aerobic and non-haemolytic corynebacteria. It displays tolerance to salts (up to 10%) and is related to the soil bacterium *Corynebacterium halotolerans*. As this is a type strain in a subgroup of *Corynebacterium* without complete genome sequences, this project, describing the 2.78 Mbp long chromosome and the 45.97 kbp plasmid pCmaris1, with their 2,584 protein-coding and 67 RNA genes, will aid the *** G****enomic* ***E****ncyclopedia of* ***Bacteria**** and* ***Archaea***** project.

## Introduction

Strain Coryn-1^T^ (= DSM 45190^T^) is the type strain of the species *Corynebacterium maris* originally isolated from the mucus of the coral *Fungia granulosa* from the Gulf of Eilat (Red Sea, Israel) [1]. The genus *Corynebacterium* is comprised of Gram-positive bacteria with a high G+C content. It currently contains over 80 members [2] isolated from diverse backgrounds like human clinical samples [3] and animals [4], but also from soil [5] and ripening cheese [6].

Within this diverse genus, *C. maris* has been proposed to form a distinct lineage with *C. halotolerans* YIM 70093^T^ demonstrating 94% similarity related to the 16S rRNA gene sequences [1]. Similar to the closest phylogenetic relative *C. halotolerans*, which displays the highest resistance to salt described for the genus *Corynebacterium* to date, *C. maris* Coryn-1^T^ is able to live under conditions with high salinity. This species grows on LB agar plates with salinity ranging between 0 and 10%. Optimal growth was detected between 0.5 and 4.0% [1]. Aside from this Coryn-1^T^ is an alkaline-tolerant bacterium, which grows well at pH 7.2-9.0 (optimum pH 7.2) [1].

Here we present a summary classification and a set of features for *C. maris* DSM 45190^T^, together with the description of the genomic sequencing and annotation.

## Classification and features

A representative genomic 16S rRNA sequence of *C. maris* DSM 45190^T^ was compared to the Ribosomal Database Project database [7] confirming the initial taxonomic classification. *C. maris* shows highest similarity to *C. halotolerans* (94%). Because sequence similarity greater than 97% was not obtained with any member of the genus *Corynebacteria*, it was suggested that *C. maris* forms an new novel species, a hypothesis that is backed by other taxonomic classifiers [1].

**Figure 1:**
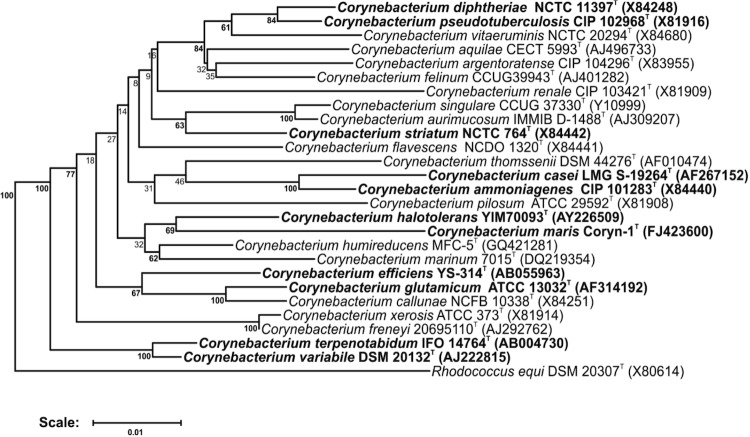
Phylogenetic tree highlighting the position of *C. maris* relative to type strains of other species within the genus *Corynebacterium*. Species with at least one publicly available genome sequence (not necessarily the type strain) are highlighted in **bold face**. The tree is based on sequences aligned by the RDP aligner and utilizes the Jukes-Cantor corrected distance model to construct a distance matrix based on alignment model positions without alignment inserts, using a minimum comparable position of 200. The tree is built with RDP Tree Builder, which utilizes Weighbor [8] with an alphabet size of 4 and length size of 1,000. The building of the tree also involves a bootstrapping process repeated 100 times to generate a majority consensus tree [9]. *Rhodococcus equi* (X80614) was used as an outgroup.

Figure 1 shows the phylogenetic neighborhood of *C. maris* in a 16S rRNA based tree. Within the larger group containing furthermore the species *C. marinum* 7015^T^ [10] and *C. humireducens* MFC-5^T^ [11], the two strains *C. maris* and *C. halotolerans* YIM 70093^T^ [1] were clustered in a common subgroup.

**Figure 2:**
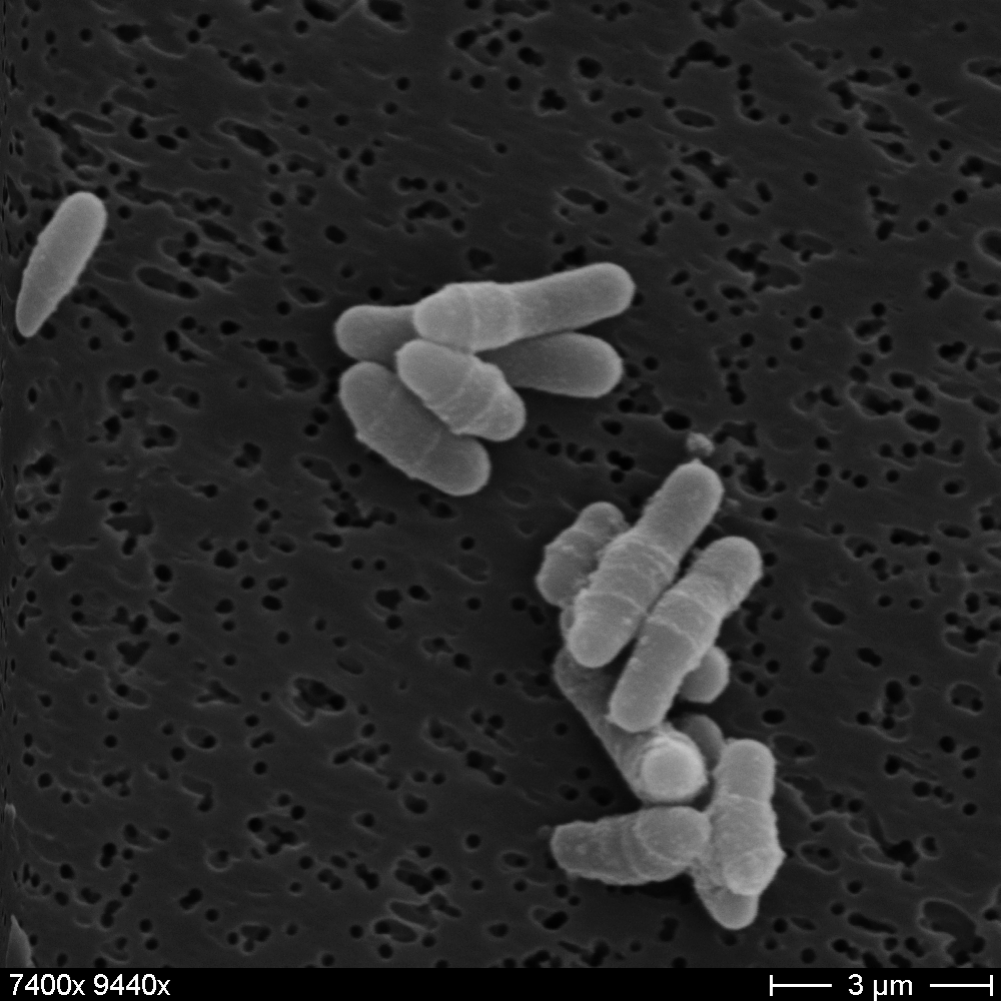
Scanning electron micrograph of *C. maris* Coryn-1^T^.

*C. maris* Coryn-1^T^ is a Gram-positive coccobacillus, which is 0.8-1.5 μm long and 0.5-0.8 μm wide (Table 1, Figure 2). By reason that *C. maris* contains a thick peptidoglycan layer, the cells commonly do not separate after cell-division and stay diplo-cellular [1], the so called snapping division.

**Table 1:** Classification and general features of *C. maris* Coryn-1^T^ according to the MIGS recommendations [12].

**MIGS ID**	**Property**	**Term**	**Evidence code^a)^**
	Current classification	Domain *Bacteria*	TAS [13]
		Phylum *Actinobacteria*	TAS [14]
		Class *Actinobacteria*	TAS [15]
		Order *Actinomycetales*	TAS [15-18]
		Family *Corynebacteriaceae*	TAS [15-17,19]
		Genus *Corynebacterium*	TAS [17,20,21]
		Species *Corynebacterium maris*	TAS [1]
		Type-strain Coryne-1^T^ (=DSM 45190^T^)	TAS [1]
	Gram stain	Positive	TAS [1]
	Cell shape	Coccus-shaped	TAS [1]
	Motility	non-motile	TAS [1]
	Sporulation	non-sporulating	TAS [1]
	Temperature range	Mesophile	TAS [1]
	Optimum temperature	35 °C	TAS [1]
	Salinity	0-10% (w/v) NaCl or sea-salt mixture (instant ocean)	TAS [1]
MIGS-22	Oxygen requirement	aerobic	TAS [1]
	Carbon source	maltose, lactulose, *β-*hydroxybutyric acid, *α*-ketovaleric acid, Tween 40, phenylethylamine, *N*-acetyl-d-galactosamine, malonic acid, l-threonine, l-glutamic acid, l-fucose, l-alanyl glycine, inosine, raffinose, d-arabitol, l-asparigine and citric acid	TAS [1]
	Energy metabolism	chemoorganoheterotrophic	TAS [1]
	Terminal electron acceptor	oxygen	NAS
MIGS-6	Habitat	mucus of the Scleractinian coral *Fungia granulosa*	TAS [1]
MIGS-15	Biotic relationship	symbiotic	TAS [1]
MIGS-14	Pathogenicity	non-pathogenic	NAS
	Biosafety level	1	NAS
MIGS-23.1	Isolation	agarsphere culturing technique	TAS [1]
MIGS-4	Geographic location	Gulf of Eilat, Red Sea, Israel	TAS [1]
MIGS-5	Sample collection time	not reported	
MIGS-4.1	Latitude	N 29°51’	
MIGS-4.2	Longitude	E 34° 94’	TAS [1]
MIGS-4.3	Depth	10-15 m	TAS [1]
MIGS-4.4	Altitude	not reported	

a) Evidence codes - TAS: Traceable Author Statement (i.e., a direct report exists in the literature); NAS: Non-traceable Author Statement (i.e., not directly observed for the living, isolated sample, but based on a generally accepted property for the species, or anecdotal evidence). These evidence codes are from the Gene Ontology project [22].

It is described as non-motile [1], which coincides with a complete lack of genes associated with ‘cell motility’ (functional category N in COGs table).

Optimal growth of Coryn-1^T^ was shown between 0.5 and 4.0% (w/v) salinity (NaCl or sea-salt mixture); however, ranges between 0 and 10% salinity are accepted [1]. *C. maris* grows at temperatures between 26-37 °C (optimum at 35 °C). Carbon sources utilized by strain Coryn-1^T^ include maltose, lactulose, *β-*hydroxybutyric acid, *α*-ketovaleric acid, Tween 40, phenylethylamine, *N*-acetyl-d-galactosamine, malonic acid, l-threonine, l-glutamic acid, l-fucose, l-alanyl glycine, inosine, raffinose, d-arabitol, l-asparigine and citric acid were used weakly [1].

Coryn-1^T^ is susceptible to sulfamethoxazole/trimethoprim, tetracycline, chloramphenicol, erythromycin, ampicillin and meticillin. The strain is resistant to nalidixic acid [1].

### Chemotaxonomy

In *C. maris* cellular fatty acids are composed of 58% oleic acid (C_18:1_ω9c), 30% palmitic acid (C_16:0_) and 12% tuberculostearic acid 10-methyl (C_18:0_). The mycolic acids of *C. maris* are short-chained, like many but not all corynemycol acids (6% C_30_, 27% C_32_, 47% C_34_ and 20% C_36_).

The biochemical characterization by Ben-Dov *et al.* [1] revealed positive signals for the following enzymes/reactions: alkaline phosphatase, esterase (C4), esterase lipase (C8), lipase (C14), leucine arylamidase, *α*-glucosidase, pyrazinamidase, pyrrolidonyl arylamidase, and gelatin hydrolysis activities.

## Genome sequencing and annotation

### Genome project history

Because of its phylogenetic position and interesting capabilities, i.e. high salt tolerance, *C. maris* Coryn-1^T^ was selected for sequencing as part of a project to define the core genome and pan genome of the non-pathogenic corynebacteria. While not being part of the *** G****enomic* ***E****ncyclopedia of* ***Bacteria**** and* ***Archaea***** (GEBA) project [23], sequencing of the type strain will nonetheless aid the GEBA effort. The genome project is deposited in the Genomes OnLine Database [24] and the complete genome sequence is deposited in GenBank. Sequencing, finishing and annotation were performed by the Center of Biotechnology (CeBiTec). A summary of the project information is shown in Table 2.

**Table 2:** Genome sequencing project information

**MIGS ID**	**Property**	**Term**
MIGS-31	Finishing quality	Finished
MIGS-28	Libraries used	Nextera DNA Sample Prep Kit
MIGS-29	Sequencing platforms	Illumina MiSeq
MIGS-31.2	Sequencing coverage	56.45×
MIGS-30	Assemblers	Newbler version 2.6
MIGS-32	Gene calling method	GeneMark, Glimmer
	INSDC ID	CP003924, CP003925
	GenBank Date of Release	July 30, 2013
	GOLD ID	Gi20930
	NCBI project ID	172964
MIGS-13	Source material identifier	DSM 45190
	Project relevance	Industrial, GEBA

### Growth conditions and DNA isolation

*C. maris* strain Coryn-1^T^, DSM 45190, was grown aerobically in LB broth (Carl Roth GmbH, Karlsruhe,Germany) at 37 °C. DNA was isolated from ~ 10^8^ cells using the protocol described by Tauch *et al*. 1995 [25].

### Genome sequencing and assembly

A WGS library was prepared using the Illumina-Compatible Nextera DNA Sample Prep Kit (Epicentre, WI, U.S.A) according to the manufacturer's protocol. The library was sequenced in a 2 × 150 bp paired read run on the MiSeq platform, yielding 1,238,702 total reads, providing 56.45× coverage of the genome. Reads were assembled using the Newbler assembler v2.6 (Roche). The initial Newbler assembly consisted of 26 contigs in seven scaffolds. Analysis of the seven scaffolds revealed one to be an extrachromosomal element (plasmid pCmaris1), five to make up the chromosome with the remaining one containing the four copies of the RRN operon which caused the scaffold breaks. The scaffolds were ordered based on alignments to the complete genome of *C. halotolerans* [26] and subsequent verification by restriction digestion, Southern blotting and hybridization with a 16S rDNA specific probe.

The Phred/Phrap/Consed software package [27-30] was used for sequence assembly and quality assessment in the subsequent finishing process. After the shotgun stage, gaps between contigs were closed by editing in Consed (for repetitive elements) and by PCR with subsequent Sanger sequencing (IIT Biotech GmbH, Bielefeld, Germany). A total of 67 additional reactions were necessary to close gaps not caused by repetitive elements.

### Genome annotation

Gene prediction and annotation were done using the PGAAP pipeline [31]. Genes were identified using GeneMark [32], GLIMMER [33], and Prodigal [34]. For annotation, BLAST searches against the NCBI Protein Clusters Database [35] are performed and the annotation is enriched by searches against the Conserved Domain Database [36] and subsequent assignment of coding sequences to COGs. Non-coding genes and miscellaneous features were predicted using tRNAscan-SE [37], Infernal [38], RNAMMer [39], Rfam [40], TMHMM [41], and SignalP [42].

## Genome properties

**Figure 3:**
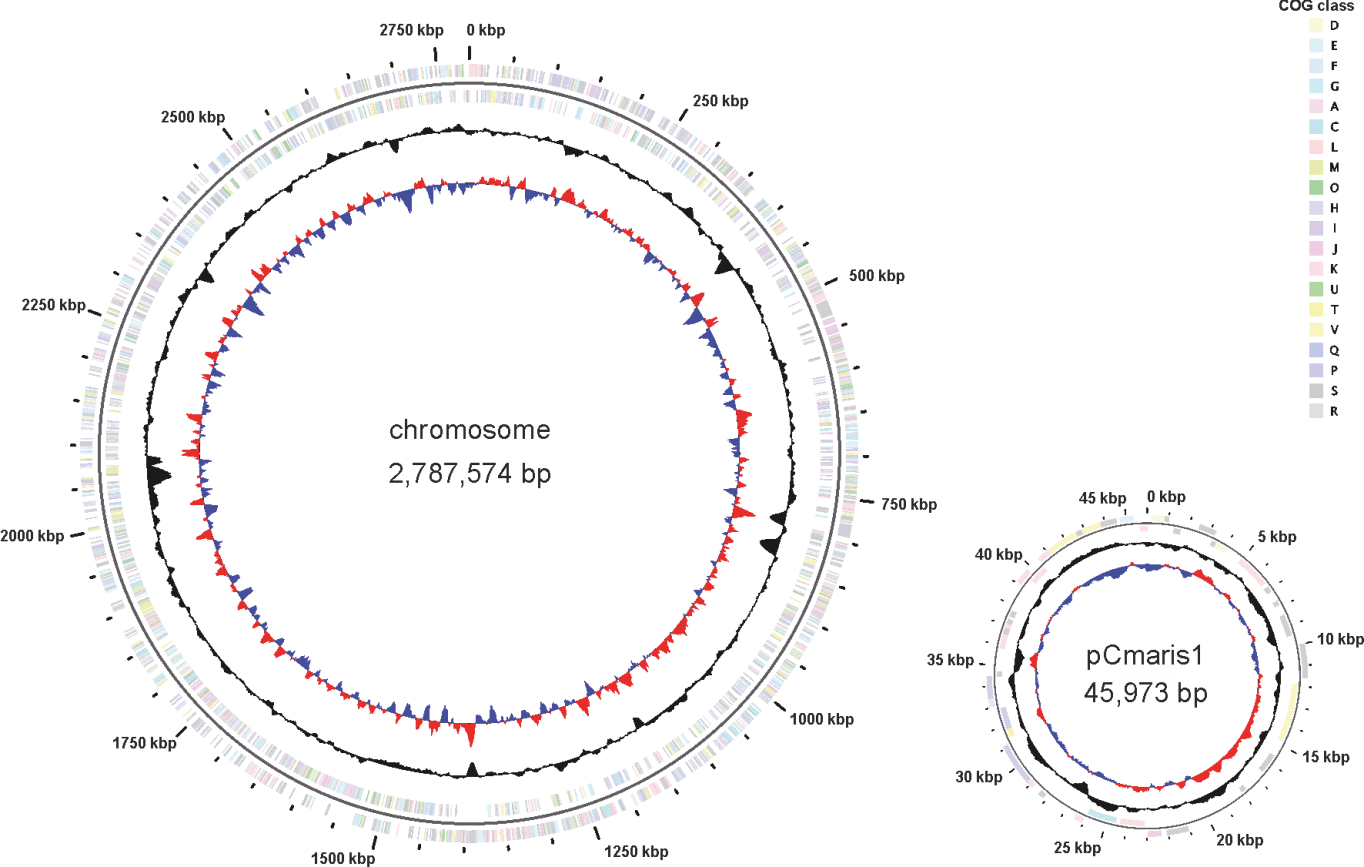
Graphical map of the chromosome and plasmid pCmaris1 (not drawn to scale). From the outside in: Genes on forward strand (color by COG categories), Genes on reverse strand (color by COG categories), GC content, GC skew.

The genome (on the scale of 2,833,547 bp) includes one circular chromosome of 2,787,574 bp (66.67% G+C content) and one plasmid of 45,973 bp (61.32% G+C content, [Figure 3]). For chromosome and plasmid, a total of 2,653 genes were predicted, 2,584 of which are protein coding genes. The remaining were annotated as hypothetical proteins. A total of 1,494 (57,82%) of the protein coding genes were assigned to a putative function. Of the protein coding genes, 1,067 belong to 350 paralogous families in this genome corresponding to a gene content redundancy of 41.29%. The properties and the statistics of the genome are summarized in Tables 3 and 4.

**Table 3:** Genome Statistics

**Attribute**	**Value**	**% of total^a^**
Genome size (bp)	2,833,547	100.00
DNA Coding region (bp)	2,508,355	88.52
DNA G+C content (bp)	1,886,661	66.58
Total genes	2,653	100.00
RNA genes	67	2.53
rRNA operons	4	
tRNA genes	55	2.07
Protein-coding genes	2,584	97.40
Genes with function prediction (protein)	1,494	57.82
Genes assigned to COGs	1,997	75.27
Genes in paralog clusters	1,067	41.29
Genes with signal peptides	226	9.54
Genes with transmembrane helices	657	24.76

a) The total is based on either the size of the genome in base pairs or the total number of total genes in the annotated genome.

**Table 4:** Number of genes associated with the general COG functional categories

**Code**	**value**	**%age**	**Description**
J	154	5.96	Translation, ribosomal structure and biogenesis
A	1	0.04	RNA processing and modification
K	163	6.31	Transcription
L	122	4.72	Replication, recombination and repair
B	0	0.00	Chromatin structure and dynamics
D	22	0.85	Cell cycle control, cell division, chromosome partitioning
Y	0	0.00	Nuclear structure
V	44	1.70	Defense mechanisms
T	63	2.44	Signal transduction mechanisms
M	116	4.49	Cell wall/membrane biogenesis
N	0	0.00	Cell motility
Z	0	0.00	Cytoskeleton
W	0	0.00	Extracellular structures
U	21	0.81	Intracellular trafficking and secretion, and vesicular transport
O	77	2.98	Posttranslational modification, protein turnover, chaperones
C	155	6.00	Energy production and conversion
G	154	5.96	Carbohydrate transport and metabolism
E	230	8.90	Amino acid transport and metabolism
F	70	2.71	Nucleotide transport and metabolism
H	111	4.30	Coenzyme transport and metabolism
I	90	3.48	Lipid transport and metabolism
P	185	7.16	Inorganic ion transport and metabolism
Q	75	2.90	Secondary metabolites biosynthesis, transport and catabolism
R	295	11.42	General function prediction only
S	181	7.00	Function unknown
-	587	22.72	Not in COGs
